# Remission of Dowager’s hump by manipulative correction of spinal alignment: a case report

**DOI:** 10.25122/jml-2023-0026

**Published:** 2023-06

**Authors:** Cherie Chau, Eric Chun-Pu Chu, Kevin Hsu-Kai Huang, Damien Tam, Gordon Cheung

**Affiliations:** 1 Mctimoney College of Chiropractic, Ulster University, Belfast, United Kingdom; 2 New York Chiropractic and Physiotherapy Centre, Hong Kong, China

**Keywords:** cervicalgia, cervicothoracic junction, chiropractic therapy, Dowager’s hump, forward head posture, myofascial trigger points, postural hyperkyphosis, thoracic spine, WHO-QOL: World Health Organization Quality of Life

## Abstract

Dowager’s hump is described as excessive kyphotic curvature in the thoracic spine with a Cobb angle of more than 40 degrees. This case report presents a 61 years old female office clerk who experienced headaches and neck pain for 3 years that extended into her right shoulder and upper chest. She consulted her primary care physician two months before seeing the chiropractor when the neck pain worsened. A diagnosis of cervicalgia related to osteoarthritis was made based on cervical and thoracic X-ray findings. The patient received non-steroid anti-inflammatory drugs (celecoxib and etoricoxib) and stretching exercises at home. At the onset of chiropractic care, radiographs showed loss of cervical lordosis, narrowing at the C4-5, C5-C6, and C6-7 intervertebral disc space with marginal osteophytes. Based on these findings, a working diagnosis of cervicogenic headache was established. After treatment for 9 months, the patient showed improvement in symptoms and function from cervical curve radiographic change and dextro-convexity of the thoracic spine. Avoiding forward head flexion and maintaining correct posture in daily activities will be key mechanisms to prevent the reoccurrence of Dowager’s hump. The improvement of symptoms following chiropractic therapy has been shown to correlate with radiographic markers of spinal realignment.

## INTRODUCTION

Dowager’s hump is described as excessive kyphotic curvature in the thoracic spine with a Cobb angle of more than 40 degrees. Patients often appear to have a bump at the base of their necks. From ages 20 to 40, the kyphosis angle averages between 20° and 29°; however, it can begin to increase significantly in both men and women after 40 years of age, with a mean of 43° in females aged 55-60 going up to 52° in those aged 76-80. Research has reported varying incidences of this condition, from twenty to forty percent within both genders [1]. It can arise due to muscle weakness or eroding sensory deficits, which can worsen over time and present the risk of vertebral fractures [2]. If left insufficiently treated, Dowager’s hump can cause functional limitations, musculoskeletal alterations, increased risk of falls, poorer satisfaction with health, and disturbances in family relationships, economic conditions, and overall quality of life [3]. Although Dowager’s hump is commonly seen in patients with hyperkyphosis, a search conducted on February 14, 2023, using the search terms "dowager's hump," "physiotherapy," and "chiropractor" in Google Scholar, Scopus, and PubMed yielded no previous cases of Dowager's Hump being seen by a primary care practitioner. The objective of this article is to present a case study of a 40-year-old woman who presented with a Dowager's Hump, along with associated neck pain and headaches. After seeking care from a chiropractor, the patient was diagnosed with hyperkyphosis of the thoracic spine and cervicogenic headache. Chiropractic professionals are healthcare practitioners who offer cost-effective management of neuromusculoskeletal disorders [4] and provide extensive treatment in spinal correction [5]. The case involves a patient who experienced neck pain exacerbated by prolonged use of a mobile device, which has been known to exert prolonged neck flexion and induce improper spine curvature [6]. However, after nine months of chiropractic therapy, the patient's symptoms completely resolved. Additionally, there was a noticeable reduction in the size of the Dowager's Hump, as confirmed by radiographic findings that demonstrated improvements in cervical lordosis, thoracic kyphosis, and dextro-convexity of the thoracic spine.

## CASE PRESENTATION

A 40-year-old female office clerk presented to a chiropractor reporting headache and neck pain lasting for 3 years. The patient reported bilateral upper trapezius pain, with greater severity on the right side, which extended into the right scapula and upper chest area. She described the pain as a persistent ache, rating it 7 out of 10 on the Numeric Pain Rating Scale. In addition, there was an exaggerated anterior curvature or hyperkyphosis of the thoracic spine.

Her symptoms started as weakness in the upper limbs and headaches that began at the base of her skull and radiated up the right side behind her ear during the first year. In the second year, she occasionally experienced visual disturbance, hand numbness, heartburn, shortness of breath, heart palpitations, and muscle cramps. The patient recently noticed that prolonged reading or using a mobile device in one position for more than 30 minutes increased muscle stiffness and difficulty in moving her neck. She had a medical history of hypertension, but she denied any history of trauma, fever, mental diseases, or loss of bowel or bladder movement. Her overall quality of life, as assessed by the World Health Organization Quality of Life (WHO-QOL) questionnaire, was rated at 72%. She initially took over-the-counter pain medications, but they did not alleviate her symptoms. She then consulted with her primary care doctor 2 months prior to seeing the chiropractor when the neck pain worsened. Cervical and thoracic radiographs revealed degenerative changes in the cervical spine, and she was diagnosed with cervicalgia related to osteoarthritis. She was prescribed non-steroid anti-inflammatory drugs (celecoxib and etoricoxib) and stretching exercises to conduct at home, but none of these treatments provided relief.

**Figure 1:**
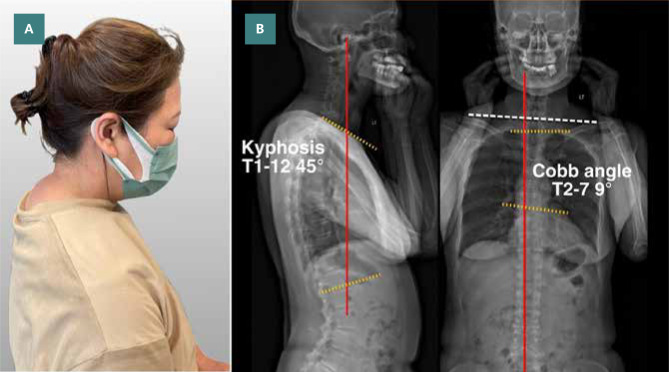
A: Posture photography identifies a Dowager’s hump appearing at the cervicothoracic junction. B: Postural analysis utilizing full-spine EOS® radiography in a standing position also showed uneven shoulders, hyperkyphosis of cervical and thoracic curves, and dextro-convexity of the thoracic spine were measured before therapy. The global axis is shown by the central sacral vertical line (red line in frontal view), and the gravity line (red line in lateral views) emphasizes the center of gravity. Kyphosis Cobb angle is 45° (normal 20°–40°), and the Cobb angle is 9°. Kyphosis Cobb angle is calculated on the lateral thoracic radiograph by drawing lines along the upper endplate of T1 and lower endplates of the T12 vertebrae. Scoliotic Cobb angle is also measured on the anteroposterior view of the spinal radiograph by drawing lines along the upper endplate of T2 and lower endplates of the T7 vertebrae. These are the most commonly accepted methods of measuring kyphosis and scoliosis and help diagnose the condition's severity and the need for treatment.

Upon visual inspection, the patient exhibited a forward head position and hunchback (thoracic hyperkyphosis) posture, where the upper back is pronouncedly shifted forward, with an elevated left shoulder. A Dowager’s hump appeared at the cervicothoracic junction (Figure 1). She was unable to maintain her entire spine in contact with the wall while standing up with her back to the wall. Myofascial trigger points and tight muscles were palpated at the deep upper cervical extensors, shoulder protractors and elevators, upper abdominals, anterior intercostal, and latissimus dorsi muscles, with greater prominence on the right side. Her range of motion of the cervical spine was restricted at 15° extension (normal >50°) and restricted at 50° of bilateral rotation (normal >80°). Motion palpation revealed intersegmental dysfunction at the C2-3, C5-6, T1-2, T2-3, and T6-7 levels. Her neurological examination was unremarkable, including an assessment of cranial nerves, sensory and motor systems, reflexes, balance and coordination, and gait. Spinal radiography identified a loss of cervical lordosis, narrowing at the C4-5, C5-C6, and C6-7 intervertebral disc space with marginal osteophytes and dextro-convexity of the thoracic spine. The Kyphosis angle was 45° (normal 20°–40°) [7], and the Cobb angle was 9°. Based on the clinical presentation and radiological findings, the patient's differential diagnosis included cervical rib, thoracic outlet syndrome, hyperkyphosis of the thoracic spine, and cervicogenic headache. Chiropractic manipulation therapy was recommended to correct the spinal alignment and stabilize the position of the cervical spine, providing an initial working diagnosis of hyperkyphosis of the thoracic spine and cervicogenic headache.

**Figure 2:**
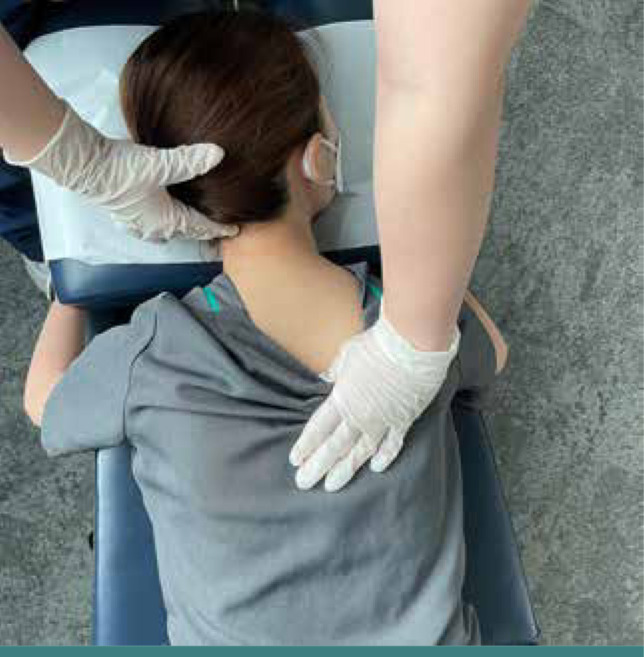
Spinal manipulative therapy: The chiropractor stabilizes the patient's head against the headrest and provides a high-velocity, low-amplitude force directed posterior to the anterior at the T7/8 articulations using the palm

**Figure 3:**
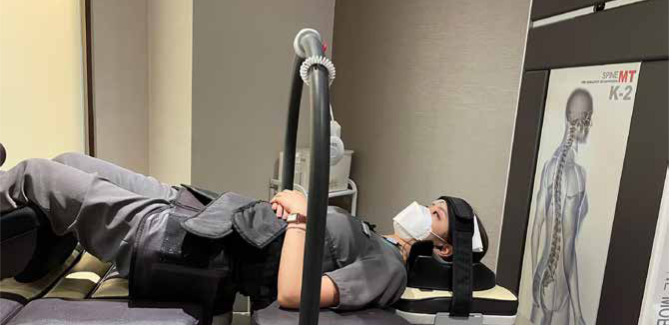
Mechanical cervical spine traction was performed on the patient in a supine position (Spine MT, Shinhwa Medical, Korea) with thoracic extension

**Figure 4:**
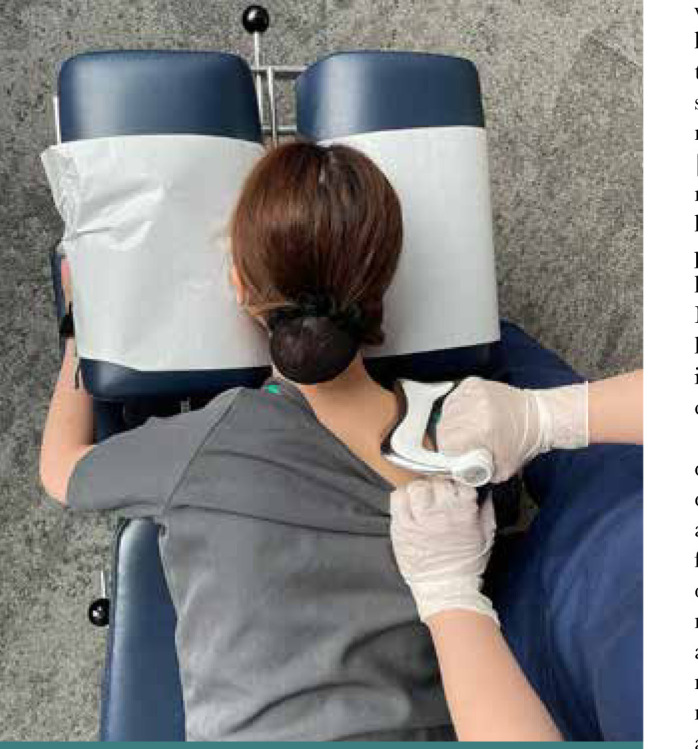
Scraping therapy: the chiropractor gently scrapes the massage advice (Strig, Korea) along the hypertonic muscles

Chiropractic therapy consisted of the following: spinal manipulative therapy (Figure 2), cervical extension-compression traction (Figure 3), and Guasha (instrument-assisted soft tissue mobilization) (Figure 4). Previous studies have demonstrated the effectiveness of spinal manipulative therapy, specifically the diversified technique, in restoring shoulder imbalance and spinal alignment [8]. Therefore, in this case, spinal manipulative therapy using the diversified technique was applied to the spinal levels exhibiting intersegmental dysfunction. Cervical extension-compression traction with CBP technique (iTrac^®^ Spine Remodelling System, USA) and mechanical cervical distraction (SpineMT, Shinhwa Medical, Korea) were applied to correct the cervical lordosis. CBP technique is widely used in correcting thoracic hyperkyphosis [9]. A glide action massage tool (Strig, Korea) was used to relieve muscular myofascial trigger points on the hypertonic muscles around the shoulder and balance shoulder height. Twelve sessions of therapy were applied in the first month. Ergonomics recommendations, such as an office workstation set-up and core-muscle exercises, were also advised to improve the forward head posture and hyperkyphosis and increase lumbar lordosis to compensate for the postural change.

**Figure 5:**
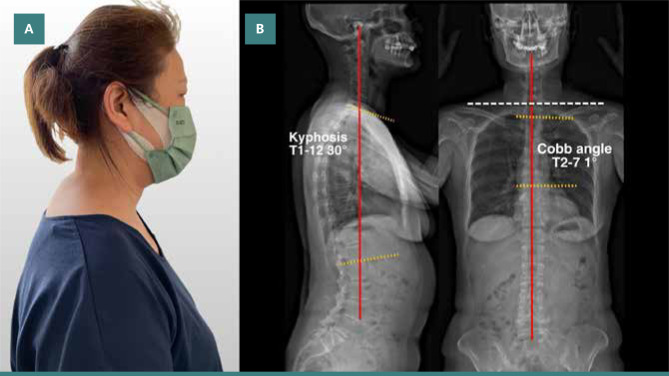
A: During re-evaluation 9 months later, the size of the hump was diminished after treatment. B: EOS® radiography showed posture balance with improved shoulder balance, central sacral vertical line, gravity line, kyphosis angle, and scoliosis measurement of Cobb angle. The kyphosis reduced from 45° to 30° and the Cobb angle reduced from 9° to 1°

By the end of the initial month of treatment, the patient's pain rating decreased from 7 to 3 on a numeric pain rating scale, and the size of Dowager's hump was diminished (Figure 5 A-B). The frequency of her therapy visits was lowered to eight sessions per month for 2 months. At the end of the third month of treatment, the patient reported that her headache entirely subsided. The therapy was continued twice per month to rectify the patient's spinal imbalances. Her headache remained absent at the 9-month follow-up visit, and her cervical range of motion had returned to normal bilaterally. Her WHO-QOL was measured at 100%. All radiological parameters greatly improved, including cervical lordosis and dextro-convexity of the thoracic spine (Figure 5).

## DISCUSSION

Excessive thoracic kyphosis is usually associated with shoulder muscle imbalance and malalignment in surrounding joints [10,11], which entails pectoralis and upper trapezius muscles tightness, along with lower trapezius and cervical flexors muscles weakness, that may increase shoulder joint pain [12]. Structurally speaking, excessive thoracic kyphosis alters shoulder position to forward tilting and protraction [13], thus leading to forward shoulder posture and scapula anteversion, resulting in impingement of the subacromial space [1] and shoulder joint dysfunction [13]. Excessive thoracic kyphosis may cause pain and compromise function in the shoulders, pelvis girdle, neck, and upper and lower back. Other signs of hyperkyphosis include forward head posture, scapula protraction, reduced standing height, decreased lumbar lordosis [13], and increased incidence of future fractures. Research has shown that older females with excessive thoracic kyphosis have a 70% higher risk of fractures than those without it. Furthermore, this risk increases as the severity of kyphosis increases [14].

Osteoporosis is commonly seen in women [15], and it has been demonstrated that women with excessive thoracic kyphosis can experience a myriad of physical impairments, such as imbalance, poor gait speed, and greater postural sway in osteoporotic females [16], thus increasing falling risks [17,18]. Impairment of upright postural control is attributed in part to declining somatosensory input from the joints in elderly adults [19-21] as well as age-related deterioration in the visual system [22]. Cataracts, macular degeneration, and deteriorating visual acuity can ultimately negatively affect one's ability to perform daily activities and overall quality of life. Excessive thoracic kyphosis posture has been attributed to higher mortality alongside an increased risk of pulmonary death [23,24]. Spinal structure improvements have been reported for symptom relief, including structure change [25], balancing and gait [26], visual disturbance [27, 28], and quality of health [29].

In this particular case, the patient received spinal manipulative therapy and extension-compression traction as part of the treatment plan. Following the chiropractic session, the patient experienced noticeable improvements in her condition. Additionally, to address the cervical lordosis and correct the altered posture, the patient was temporarily positioned in a cervical extension posture. Thoracic spinal manipulation and extension-compression traction improved thoracic hyperkyphosis [30,31]. Additionally, enhancing the middle and lower trapezius minimize protraction of scapulars [32], which gives better forward shoulder posture. Certain neck and craniofacial areas may have myofascial trigger points that aggravate the discomfort and foster cervicogenic headache [33]. Reported findings suggest that myofascial trigger points in the suboccipital, sternocleidomastoid, and upper trapezius muscles are associated with painful conditions [34,35]. Scraping therapy utilizes an instrument to apply pressure or scrape the skin, which helps increase the blood flow in the area with potential benefits on myofascial trigger points, neck pain, and cervicogenic headache [36]. A large study found that chiropractic therapy has rare adverse events [37] and significant benefits to musculoskeletal complaints. Our case study further supports these findings by demonstrating the successful reduction of Dowager's hump through manipulative correction of spinal alignment. However, it is important to acknowledge that this case study has limitations in terms of its generalizability to the broader population, as it provides limited evidence for extrapolating outcomes.

## CONCLUSION

The current study reported a case of Dowager’s hump or excessive kyphotic curvature in the thoracic spine. The improvement of symptoms and range of motion following chiropractic therapy has been shown to correlate with radiographic markers of spinal realignment. Excessive kyphotic curvature in the thoracic spine can lead to various functional limitations and musculoskeletal alterations and affect the overall quality of life. Patients’ education in maintaining correct posture in daily activities is crucial to prevent the recurrence of Dowager’s hump.

## Data Availability

Further data are available from the corresponding author upon reasonable request.
